# Cancer immune evasion through KRAS and PD-L1 and potential therapeutic interventions

**DOI:** 10.1186/s12964-023-01063-x

**Published:** 2023-03-02

**Authors:** Alex Watterson, Matthew A. Coelho

**Affiliations:** 1 Translational Cancer Genomics, Wellcome Sanger Institute, Hinxton, UK; 2 Open Targets, Cambridge, UK

## Abstract

Oncogenic driver mutations have implications that extend beyond cancer cells themselves. Aberrant tumour cell signalling has various effects on the tumour microenvironment and anti-tumour immunity, with important consequences for therapy response and resistance. We provide an overview of how mutant RAS, one of the most prevalent oncogenic drivers in cancer, can instigate immune evasion programs at the tumour cell level and through remodelling interactions with the innate and adaptive immune cell compartments. Finally, we describe how immune evasion networks focused on RAS, and the immune checkpoint molecule PD-L1 can be disrupted through therapeutic intervention, and discuss potential strategies for combinatorial treatment.

**Video abstract**

**Supplementary Information:**

The online version contains supplementary material available at 10.1186/s12964-023-01063-x.

## Introduction

As our understanding of cancer and the tumour microenvironment (TME) has improved, more advanced targeted and refined therapies have been developed, including cancer immunotherapies that leverage the patient’s immune system. For example, immune checkpoint blockade (ICB) is now clinically approved for the treatment of a multitude of different cancer types. Most cancer types employ some form of immune evasion [1–4]. Mechanistically, this can range from the remodelling of the TME through increased recruitment of Myeloid-Derived Suppressor Cells (MDSC) and T regulatory (Treg) cells, and stromal remodelling with TGF-β induction of Cancer-Associated Fibroblasts (CAFs), to differential cytokine (VEGF, IL-6, IL-8) and checkpoint molecule (PD-L1, B7-H4) expression. In this review, we will focus on tumour-intrinsic immune modulation through oncogenic signalling via RAS and other prominent oncogenes, and how this can affect the immune contexture [5] through differential expression of immune checkpoint molecules and other mechanisms [2, 3, 6–9]. In particular, we will discuss how our understanding of tumour cell-intrinsic KRAS signalling and PD-L1 expression has evolved in relation to anti-tumour immunity and mechanisms of immune escape, and how this is driving innovation in research and clinical settings.

### Immune evasion and oncogenic KRAS—what led us here?

**Fig. 1:**
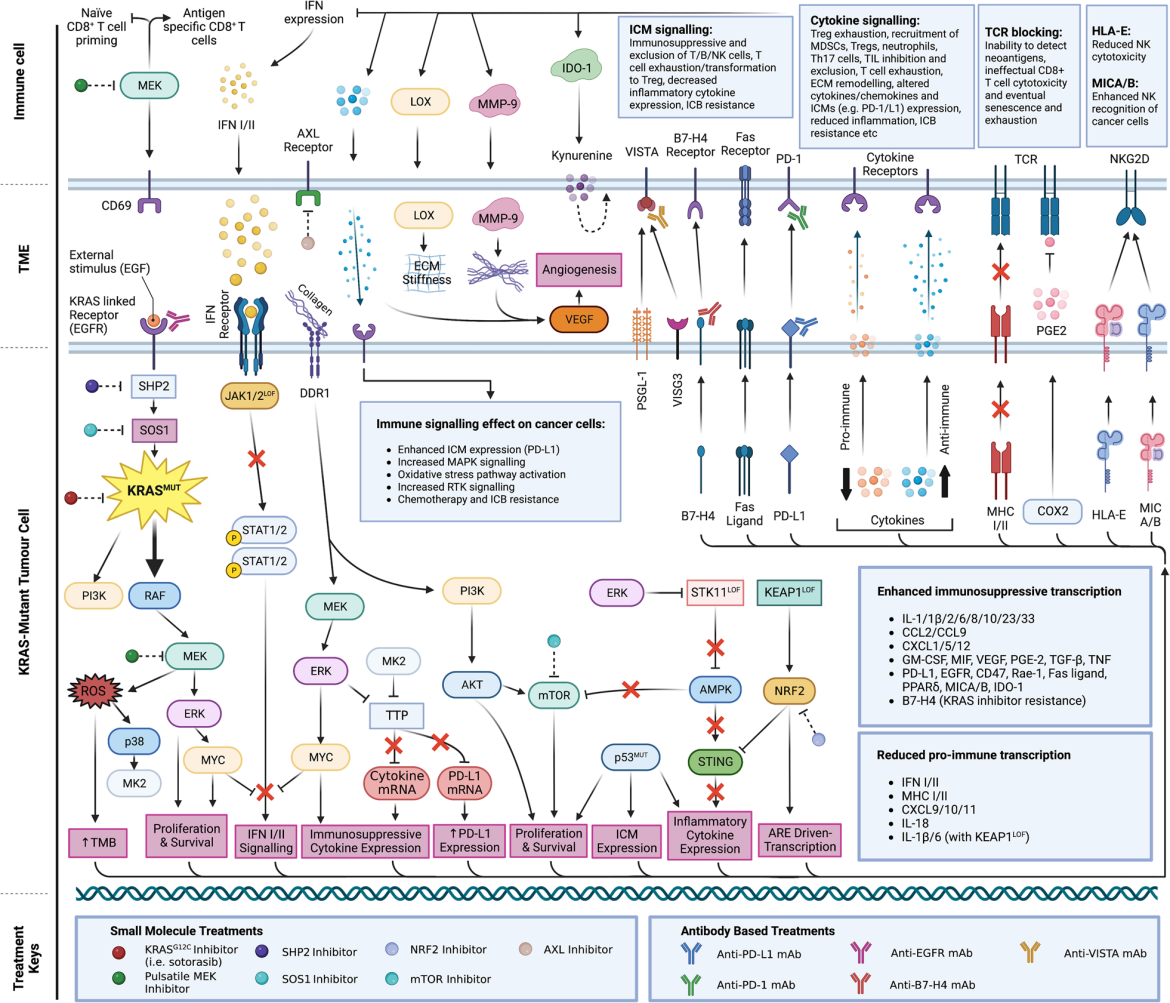
Immunoevasive signalling pathways in KRAS-mutant cancers. Immune evasion is mediated via MAPK pathway signalling enhanced by mutant KRAS, decreased type I/II IFN signalling, TTP inhibition, and co-mutations (e.g. *STK11/KEAP1/TP53/EGFR*). The effect of these pathway alterations is represented with cell-autonomous (e.g. increased survival and proliferation) and non-cell-autonomous programmes (e.g. decreased MHC expression, increased PD-L1 expression). Signalling through the TME to immune cells, via free and bound signalling molecules, and the immune evasive effects are also highlighted and attributed to specific signalling pathways (e.g. ICM suppression of CD8 + T cells, and reduced neoantigen detection due to PGE2). Immune cell metabolic reprogramming (IDO-1), increased pro-tumour cytokines and physical remodelling of the TME (MMP-9 and LOX), due to the KRAS signalling demonstrate its central role in tumour immune evasion. Treatments for abating these immune evasive mechanisms are shown. Arrows represent enhancement and flat heads represent inhibition, dotted lines are used to indicate inhibition by small molecule treatments, and red crosses represent pathways that have been blocked due to mutation or alterations in cell signalling

Mutations in *RAS* are some of the most frequent mutations across all cancer types, with a 15–30% mutation rate across all cancers [7, 9]. Alterations in the KRAS isoform account for 75–85% of *RAS*-mutant cancers [10, 11], with mutations at G12 accounting for 81% of these [11], and are mainly associated with non-small cell lung cancer (NSCLC), colorectal cancer (CRC), and pancreatic ductal adenocarcinoma (PDAC) [12]. However, *KRAS* driver mutation and allelic frequency vary between tissue types [13]. KRAS is a GTPase, with activating mutant forms of KRAS favouring the GTP-bound, active state. Such aberrant signalling leads to transcriptional upregulation of intrinsic pro-survival, anti-apoptotic, angiogenic, and proliferative pathways such as PI3K-AKT-mTOR and MAPK, as well as immune evasion mechanisms such as reduced antigen presentation (Fig. 1) [6, 7, 9, 14]. As we discuss below, oncogenic RAS has been associated with significantly enhanced immune checkpoint molecule (ICM) expression and immune evasion programmes, making *RAS*-mutant tumours promising candidates for ICB.

Recent pharmacological research has been focused on targeting the third most common G12 *KRAS* mutation, KRAS^G12C^ (11.9%), which trails behind the most common *KRAS* mutations, KRAS^G12D^ and KRAS^G12V^ (29.5% and 23%, respectively) [15]. Interestingly, the KRAS^G12C^ variant is associated with a higher response rate to ICB and progression free survival (PFS), perhaps because of a significant co-occurrence of high tumour mutational burden (TMB)/microsatellite unstable status and the potential for an “immune-hot” TME [15–17]. Indeed, in both non-small cell lung cancer (NSCLC) and colorectal cancer (CRC), high-TMB smoker patients are more likely to be KRAS^G12C^ [15]. This trend towards favourable ICB responses can also be seen with the KRAS^G12V^ variant, likely due to the commonality of the KRAS^G12C^ and KRAS^G12V^ variants attributed to smoking, and consequently a high-TMB [16]. In contrast, KRAS^G12D^, a driver in approximately 40% of PDAC tumours [18], has been noted as having a reduced response rate to immunotherapies, including ICBs [19]. This has been attributed to particularly high levels of GM-CSF production [20], a high degree of mutational intertumoral and intratumoral heterogeneity [21], most KRAS^G12D^-driven NSCLC tumours presenting in more “immune cold” never-smokers [22], and potentially, lower PD-L1 expression in KRAS^G12D^ tumours [16]. Furthermore, KRAS^Q61X^, accounting for ~ 7% of *KRAS* mutant NSCLCs [23], has been associated with decreased TMB and PD-L1 expression [24]. This is significant because the Checkmate 568 trial of NSCLC patients treated with nivolumab plus ipilimumab combination therapy confirmed that a low TMB is an important determinant of treatment failure [25]. However, other studies have found that *KRAS*-mutation subtype has no significant effect on PFS or OS, instead attributing response to other factors such as co-mutations in *TP53*, *KEAP1*, *STK11, EGFR* and *SAMARCA4* [22, 26, 27], highlighting the complexity of this disease and the need for further studies.

**Fig. 2:**
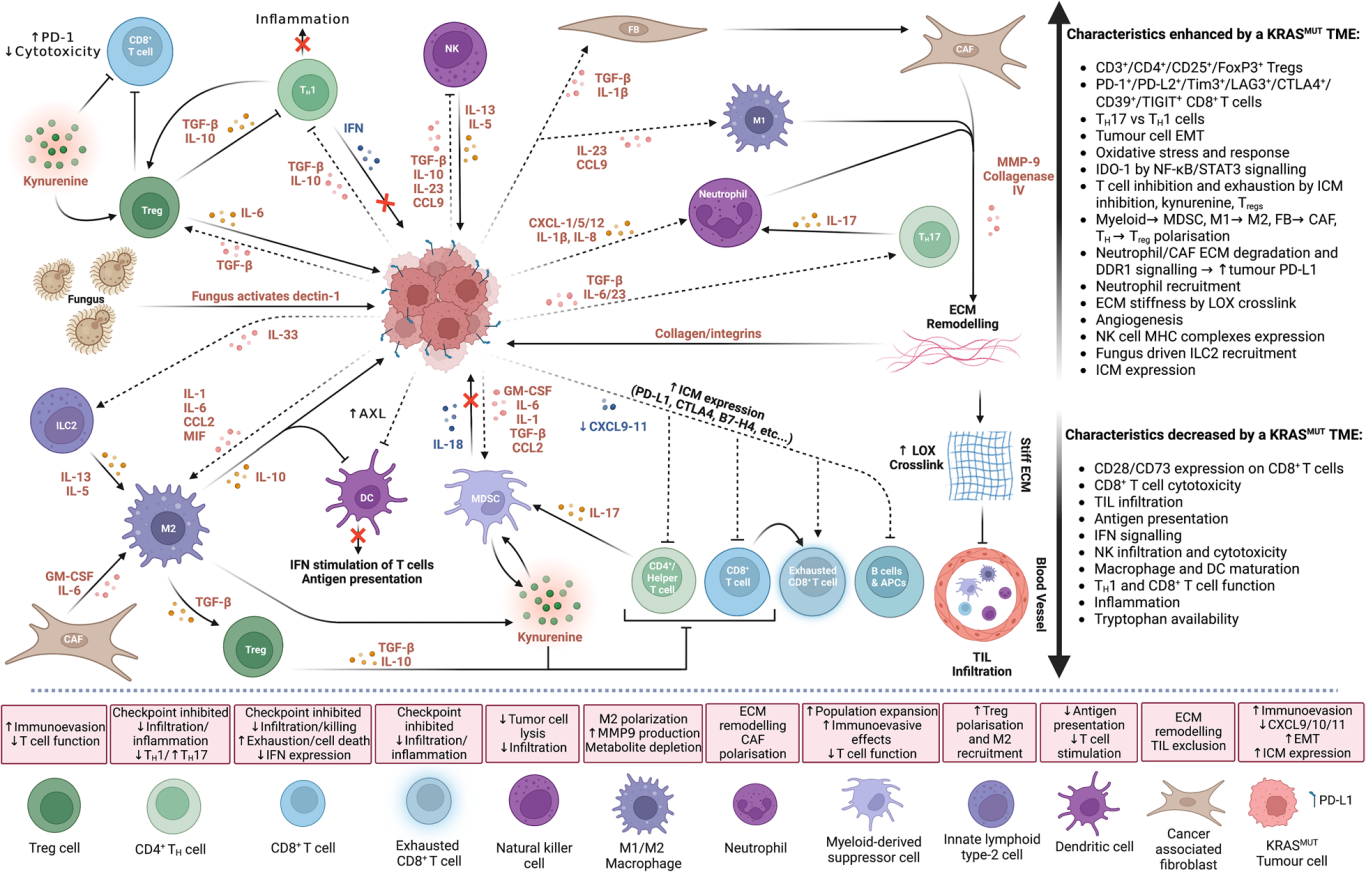
KRAS-mutant tumour immunoevasive signalling in the TME. Tumour-immune and immune-immune interactions within the TME of a KRAS-mutant tumour, including cytokines and chemokines signalling, metabolic deficiencies, immune checkpoint molecules (ICMs), interactions with the fungal microbiome, and remodelling of the extracellular matrix (ECM) and stroma. For example, the main effects of each cell type and decreased/increased characteristics of a KRAS-mutant TME are shown. Arrows represent enhancement and flat heads represent inhibition, dashed lines represent tumour signalling and hard lines represent signalling of the TME, red text indicates immunoevasive signalling and blue text indicates pro-immune signalling. Red crosses represent pathways that have been blocked due to KRAS-driven immune evasive signalling

The intrinsic and extrinsic mechanisms by which *KRAS* mutations and tumour PD-L1 expression remodel the immune landscape of the TME have become a focus of research in recent years. An interesting genetic mechanism for enhanced immune suppression was discovered, whereby 3’UTR loss in *PD-L1* (*CD274*) resulted in enhanced expression and more effective immune evasion in multiple cancers, though the precise mechanism for this upregulation was unclear at the time [28]. It was later demonstrated that oncogenic RAS signalling could drive PD-L1 expression in cancer cells. Mechanistically, enhanced *PD-L1* mRNA expression is driven through RAS-MEK signalling and the RAS-ROS-p38 axes, phosphorylating TTP and inhibiting TTP binding to AU-rich elements in the *PD-L1* mRNA 3’UTR, reducing mRNA degradation [3]. PI3K signalling downstream of RAS has also been implicated in increasing translation of the *PD-L1* mRNA transcript, overall leading to coordinate upregulation of mRNA and protein [29] (Fig. 1). Extrinsically, increased KRAS signalling and TTP inhibition have been linked to an altered cytokine/chemokine signature (e.g. IL-8, GM-CSF, CXCL5, IL-10, VEGF, PEG-2), giving rise to the recruitment of myeloid-derived suppressor cells (MDSC) and Tregs, CAF transformation, and increased endothelial Fas ligand expression, which can inhibit CD8 + T cell extravasation (Fig. 1, Fig. 2) [3, 8, 9].

Clinically, the prospect of combining targeted therapies with ICB is exciting, especially with the recent development and FDA approval of the first-in-class KRAS^G12C^-specific inhibitor, sotorasib (AMG-510) (Fig. 1) [2]. The chemically tractable mutant cysteine of G12C allows for mutant-specific covalent binding to inhibitors, concomitant with an extremely low off-target score and no inhibitory effect on wild-type KRAS or other KRAS-mutant variants [2]. Such mutant-specific compounds can lead to a step-change in both the reduction of associated toxicities and benefit for patient outcomes in KRAS^G12C^-mutant NSCLC and CRC [2]. Interestingly, sotorasib requires an adaptive immune response to provide maximum benefit [2, 7]. In responsive tumours, sotorasib has been seen to encourage a proinflammatory TME and increase T cell infiltration via increased CXCL10/11 (Fig. 1) [2, 9]. ICB is still only effective in a subset of patients [30], so these recent discoveries have provided an indication that such ICB-combination strategies could improve response rates in some contexts. Finally, a unique advantage to covalently modifying the mutant KRAS^G12C^ protein with an inhibitor is the potential to specifically target drug-modified KRAS epitopes with antibodies or cell therapies [31], precisely flagging *KRAS*-mutant tumour cells for immune-mediated destruction.

### The TME, PD-L1 and KRAS—Recent innovations in treatment and novel mechanisms

#### Novel combination therapies for the treatment of KRAS-driven malignancies

Clinical trials are now underway that combine ICB with sotorasib, MEK and ERK inhibitors [2, 8, 9]. Current trends have been focused on understanding the mutational and immune landscape of *KRAS*-mutant tumours to elucidate potential resistance mechanisms and immune modulatory niches that might be rationally exploited. Clinical trials evaluating MEK inhibition alongside PD-1/L1 therapy (Fig. 1) in *KRAS*-mutant tumours had limited success, ostensibly due to tumour heterogenicity and MEK inhibition of CD8 + T cell IL-2 production, which is crucial for clonal expansion and leads to an exhausted phenotype, inhibiting the adaptive immune response [14, 32]. Interestingly, in a colon mouse tumour model, a synergistic effect was produced by combining sotorasib alongside both trametinib (MEK inhibitor) and anti-PD-1 therapy, greatly enhancing survival [2]. There is evidence that a pulsatile dosing regimen for MEK inhibitors can alleviate the anti-proliferative effect on CD8 + T cells, actually resulting in enhanced Ki-67 and CD69 detection; hallmarks of successful clonal expansion (Fig. 1) [14]. This is in agreement with a study in mice by Ebert et al., where MEK inhibition with cobimetinib profoundly blocked naïve CD8^+^ T cell priming, but led to a greater proportion of antigen-specific CD8^+^ T effector cells within the tumour by abating chronic T cell receptor (TCR) stimulation (Fig. 1), and ultimately resulted in a synergistic effect with PD-L1 ICB [33]. Clinical trials (NCT03581487, NCT03600701) are currently recruiting to evaluate this further in NSCLC patients (Table 1).

**Table 1:** Clinical trials assessing the efficacy of combinations with immunotherapies in cancers with RAS mutations

Trial	Disease	Pathway/Target	Treatment	Status
Checkmate 568 (NCT02659059)	NSCLC	PD-1 CTLA-4 DNA cross-linking	Nivolumab Ipilimumab Platinum Doublet Chemotherapy (Carboplatin + Paclitaxel and Cisplatin + Pemetrexed)	Complete
NCT03581487	NSCLC	MAPK/MEK PD-1 CTLA-4	Selumetinib Durvalumab Tremelimumab	Recruiting
NCT03600701	NSCLC	PD-L1 MAPK/MEK	Atezolizumab Cobimetinib	Recruiting
KRYSTAL-1 (NCT03785249)	KRAS G12C Advanced/Metastatic solid tumours	KRAS G12C PD-1 MAPK/EGFR ErbB family protein kinases	MRTX849 (adagrasib) Pembrolizumab Cetuximab Afatinib	Recruiting
KRYSTAL-7 (NCT04613596)	Advanced/Metastatic NSCLC	KRAS G12C PD-1	MRTX849 Monotherapy MRTX849 in Combination with Pembrolizumab	Recruiting
CodeBreak 100 (NCT03600883)	KRAS G12C Advanced solid tumours	KRAS G12C PD-1/L1	Sotorasib Anti-PD-1/PD-L1 Midazolam	Active, not recruiting
CodeBreak 101 (NCT04185883)	KRAS G12C Advanced solid tumours	KRAS G12C PD-1 MAPK/MEK MAPK/SHP2 ErbB family protein kinases MAPK/EGFR DNA cross-linking Purine synthesis /Thymidylate synthase Microtubular depolymerization and bcl-2 gene expression PD-L1 PI3K/mTOR CDK4/6 MAPK/VEGF	Sotorasib AMG-404 Trametinib RMC-4630 Afatinib Pembrolizumab Panitumumab Carboplatin Pemetrexed Docetaxel Atezolizumab Everolimus Palbociclib MVASI (bevacizumab-awwb) TNO155 FOLFIRI FOLFOX Loperamide	Recruiting
NCT05389514	KRAS G12V Mutant Advanced Epithelial Cancers	Cross-linking of DNA DNA synthesis inhibition PD-1	Cyclophosphamide Gemcitabine Pembrolizumab Cell Infusion of T cells expressing HLA-DRB1*07:01-restricted KRAS G12V reactive T-cell receptors	Recruiting/ Available
NCT04620330	NSCLC with KRAS G12V and BRAF Activating Mutation	MAPK/Dual RAF and MEK inhibitor p53 pathway/FAK Inhibitor	VS-6766 Monotherapy VS-6766 and Defactinib in combination	Recruiting
NCT03146962	RAS or BRAF driven Colorectal, Pancreatic and Lung Cancers	Oxidative stress pathway/ NRF2	High dose Ascorbic Acid infusion	Recruiting
NCT04919369	Metastatic Lung Non-Small Cell Carcinoma Recurrent Lung Non-Small Cell Carcinoma Stage IV Lung Cancer AJCC v8 Stage IVA Lung Cancer AJCC v8 Stage IVB Lung Cancer AJCC v8	KEAP1-NRF2/tBHQ-ARE PD-L1	Tretinoin/ All-Trans Retinoic Acid (ATRA) Atezolizumab	Recruiting
NCT01347866	Advanced cancer	PI3K/mTOR MAPK/MEK Topoisomerase I	PF-05212384 PD-0325901 Irinotecan	Active, not recruiting
NCT03514121	Advanced Solid Tumours Breast Cancer Ovarian Cancer Endometrial Cancer	B7-H4 PD-1	FPA150 Pembrolizumab	Complete
NCT05082610	Metastatic and Advanced Solid Tumours in Non-small Cell Lung Cancer Triple Negative Breast Cancer Malignant Neoplasm	VISTA PD-1	HMBD-002 Pembrolizumab	Recruiting
NCT02908672	Melanoma	PD-L1 MAPK/MEK BRAF	Atezolizumab Cobimetinib Vemurafenib	Active, not recruiting

For each trial, the disease, pathway/target, treatment, and trial status are indicated

#### Mutant-specific KRAS inhibitors and anti-tumour immunity

With multiple mutant-specific KRAS^G12C^ inhibitors entering the clinic (JNJ-74699157, JDQ443, GDC-6036, and LY3499446), many clinical trials are underway to assess combinations of PD-1 pathway ICB and different KRAS^G12C^ inhibitors, spanning PDAC, NSCLC and CRC patients in varying stages (KRYSTAL-1, KRYSTAL-7, CodeBreak 100, and CodeBreak 101) (Table 1). The number of possible combinations to test clinically could rapidly expand, which emphasises the need to understand mechanisms of action on the tumour cell, and the interplay between tumour cells and the immune compartment, to identify robust biomarkers of response [15].

For example, recent work by Downward and colleagues showed that KRAS^G12C^ inhibition in a *KRAS*-mutant mouse model of NSCLC suppresses the downstream function of MYC, resulting in up-regulated interferon signalling, leading to reduced tumour immunosuppressive cytokine production, enhanced infiltration and activation of CD8 + T cells, and increased neoantigen presentation by MHCs (Fig. 1, Fig. 2) [7]. Without pharmacological intervention, aberrant KRAS and MYC programs act cooperatively to drive tumorigenesis and extensive remodelling of the TME [34, 35]. Indeed, in vivo models of cancer have also demonstrated how overactive MYC enhances the expression of the ICMs CD47 and PD-L1 [36], as well as the anti-immune signalling molecules IL-23 and CCL9 (Fig. 1, Fig. 2) [37]. Taken together, these signals can cause extensive local remodelling of both the adaptive and innate immune cell compartments through decreased MHC I expression, upregulation of Rae-1 (NKG2D receptor ligand), enhanced angiogenesis by VEGF [36], reduced macrophage recruitment, and PD-L1 dependent T/B/APC/NK cell exclusion (Fig. 1, Fig. 2) [37]. Inhibition of MYC resulted in marked apoptosis and tumour regression [36, 37]. However, combinations of KRAS^G12C^ inhibitors with PD-L1 therapy only showed synergy in highly immunogenic tumours, with many still developing secondary resistance following treatment [7, 15, 38].

Modulation of interferon signalling is crucial for *KRAS*-mutant immune evasion. Indeed, a recent study observed that when a variety of mouse KRAS^G12C^ lines (including the immune-hot KPAR and immune-cold KPB6 [39]) were treated with the KRAS^G12C^ inhibitor MRTX1257, the transcriptional effects of IFN-γ signalling were enhanced in all cell lines. This resulted in an upregulation of T cell chemo-attractants such as Cxcl9/10/11 and antigen presentation genes including H2-d/k1, Ciita, and B2m (Fig. 1) [7], thus demonstrating how even neoantigen-high *KRAS*-mutant tumours can escape the adaptive immune response [7, 8, 40].

#### Resistance mechanisms in KRAS-driven malignancies—how might these be overcome?

KRAS^G12C^ combination strategies with ICB could be helpful to forestall the onset of acquired drug resistance. Resistances to KRAS^G12C^ inhibitors include increased receptor tyrosine kinase (RTK) feedback reducing GDP occupancy [41], secondary KRAS mutations including G12D, G13, Q61, R68, H95, and Y96 mutations, or amplification of the KRAS^G12C^ allele [42]. Combinations of KRAS^G12C^ inhibitors with ICB, chemotherapy, anti-EGFR antibodies [43], and pan-KRAS targeting agents (e.g. SOS1 and SHP2 inhibitors) are being investigated (Fig. 1) [7, 44–46]. Moreover, the use of new combinations with pan-KRAS inhibitors is already being tested, as are SHP2 inhibitors (SHP2i/RG6433) and SOS1 inhibitors (BAY-293 and BI-3406) in combination with KRAS^G12C^ inhibitors [45, 46]. Additionally, trials targeting KRAS^G12V^ in advanced epithelial cancer (NCT05389514) and NSCLC (NCT04620330) are taking place with ICB and RAF/MEK inhibitors, respectively (Table 1). Zygosity and location of KRAS mutations have a large effect on KRAS-KRAS dimerization, which is crucial for KRAS downstream signalling and is involved in mechanisms of resistance [47]. Investigations into how oncogenic *KRAS* mutations and mutant-specific inhibitors affect dimerization are currently being explored as a method for reducing resistance, such as the resistance mutation KRAS^Y96D^ for sotorasib and MRTX849 treatment [48]. Indeed, resistance by *KRAS* mutational heterogenicity affecting dimerization provides a good rationale for the use of MEK inhibitors to disrupt this mechanism [47]. Additionally, resistance can be achieved through gain-of-function mutations in epigenetic regulators, producing a profound effect on signalling and tumour metabolism within the TME. Following genetic KRAS silencing, HDAC5 upregulation causes remodelling of the TME via SOCS3-dependent upregulation of CCL2 and CCL7, and recruitment of immunosuppressive tumour-associated macrophages (TAMs) (Fig. 2) [49]. This process is driven through increasing TGFβ signalling and enables KRAS-independent tumour progression [49]. These data highlight the potential for other novel co-treatments to be employed in *KRAS*-mutant cancers, which may become resistant to KRAS inhibition. For example, CCR2 inhibitors such as PF-04136309 (ORR of 40% in PDAC trials [50]) or epigenetic drugs such as HDAC inhibitors could be leveraged to impede KRAS-independent tumour progression [51].

Immunoediting of neoantigens, reduced HLA expression and inactivation of the IFN-γ pathway are perhaps the most ubiquitous examples of immune evasion in *KRAS*-mutant PD-L1 + cancers, while acquired resistance to immunotherapies tends to be more nuanced. A poignant example of this is the discovery of an on-target mutation in *PD-L1* as a result of tumour challenge with EGFR (cetuximab) and PD-L1 (avelumab) antibodies plus chemotherapy (FOLFOX) [38]. Following therapy, a truncating mutation (PD-L1^K162fs^) and a new phosphorylation site mutation (PD-L1^L88S^) emerged, leading to *PD-L1* mRNA degradation by nonsense-mediated decay, and causing loss of protein stability and proteasomal degradation, respectively [38]. Treatment cessation led to these mutant subclones declining, indicating that drug holidays may be beneficial in this context [38]. Additional subclones with loss of function (LOF) mutations in JAK proteins were also seen (Fig. 1), further implicating the role of IFN-γ and the adaptive immune response as major determinants of successful anti-tumour responses in *KRAS*-mutant malignancies [38]. More recently, it was observed that *KRAS*-mutant pancreatic tumours acquire *SMAD4*/*TGFBR2* LOF mutations (35–50%) as a resistance mechanism to ICB [52]. Compounds which bind target proteins and induce the ubiquitination and subsequent degradation through E3 ligases (Proteolysis Targeting Chimeras, PROTACs) are also being utilised to target PD-L1 in cancer [53, 54], and could serve as a useful, orthogonal treatment avenue to antibody-mediated ICB therapies.

#### Concurrent mutations with KRAS and immune evasion

Although oncogenic KRAS can orchestrate immune evasion pathways, the response of *KRAS*-mutant tumours to ICB is still low [~ 25%] [26], with perhaps the most reliable biomarker for response remaining TMB [30]. Hence, multiple studies have identified genomic and phenotypic changes accompanying innate or acquired immunoresistance. *KEAP1*, *STK11*, *LRP1B* and *CDKN2A* mutations and mismatch repair defects commonly co-occur in *KRAS*-mutant NSCLC and CRC [15, 55–58]. In addition, *KEAP1* and *STK11* LOF mutations have both been linked to immune evasion by reducing the number of tumour-infiltrating lymphocytes (TILs) within the TME (Fig. 1) [59, 60].

KEAP1^LOF^ occurs in ~ 20% of NSCLC, is associated with an immunosuppressive microenvironment, and results in increased levels of NRF2-ARE-driven expression [57, 59, 61]. NRF2 itself has been associated with decreased TILs, increased EGFR and PD-L1 expression, reduced IL-6 and IL-1β expression [62], blocking T cell receptor signalling via COX2/PGE2, and blocking cGAS/STING pathway signalling (Fig. 1) [26, 38, 39, 41, 42]. Indeed, COX2/PEG2 signalling plays an important role in sustained pro-tumour inflammatory pathways and has been observed in Braf^V600E^ and Nras^G12D^ melanoma cells, with inhibition of this pathway promoting anti-tumour immunity and tumour rejection when combined with ICB [63]. STK11^LOF^ occurs in approximately 15% of lung adenocarcinomas and is associated with a lack of tumour PD-L1 expression, enhanced mTOR activity, CD8 + T cells transformation to exhaustion phenotype and reduction of new infiltrates, neutrophil recruitment [64], blocking of cGAS/STING signalling [57, 65], and resistance to ICB in patients with *KRAS*-mutant NSCLC (Fig. 1) [57, 65, 66]. Interestingly, KEAP1^LOF^ alone increases PD-L1, but when combined with *KRAS*-mutation, PD-L1 expression is reduced, whereas *STK11* mutation is associated with decreased PD-L1 expression regardless of *KRAS* mutation status [57]. Curiously, both *KEAP1* and *STK11* mutations are associated with worse prognosis in tumours harbouring KRAS G12C mutations compared to G12V or G12D. *KEAP1* and *STK11* mutations are associated with positive survival impact in *KRAS*-WT tumours [55, 57]. This may be due to the reduced Foxp3 expression seen in STK11^LOF^/KRAS^WT^ tumours which impairs the function of T_regs_ [67], as well as the increased TMB and neoantigen load seen in both STK11^LOF^ and KEAP1^LOF^ tumours driven from aberrant ROS generation [68] and a lack of the KRAS-dependent MHC complex downregulation [34], which would enhance the effect of ICB (Fig. 1). NRF2 modulators/inhibitors (e.g. luteolin, ascorbic acid, tretinoin) might prove helpful in treating *KRAS*/*KEAP-*mutant cancers [69] (Fig. 1); 2 clinical trials are currently underway investigating the clinical efficacy of ascorbic acid (NCT03146962) and tretinoin (NCT04919369) in NSCLC and RAS-driven cancers respectively (Table 1). mTOR inhibitors (Fig. 1), while previously unsuccessful in a 2018 study in *KRAS*-mutant cancers (NCT01347866) (Table 1), have been seen to promote tumour regression in the treatment of KRAS^G12C^ inhibitor refractory disease [41], and may yet prove effective by using *STK11*-mutation status as a surrogate biomarker for pathway activity. Lastly, treatment of *Kras/Trp53/Stk11*-mutant tumours with Axl inhibitors has recently been shown to reverse STK11^LOF^-driven resistance to PD-1 ICB in mouse models through enhancing type I IFN production by DCs to promote T cell proliferation (Fig. 2) [70].

Another study analysing the potential non-cell-autonomous effects of oncogenic signalling in KRAS^G12C^-driven NSCLC found significant differentially expressed genes, mainly associated with the regulation of cell proliferation and migration, immune cells and hormone signalling [71]. The most noteworthy of these included the downregulation of *VTCN1* (B7-H4) [71]. B7-H4^+^ tumour status has been previously shown to co-express in *KRAS-*mutant NSCLC (76%) [72] and other *KRAS-*mutant cancers such as CRC [73]. B7-H4 acts to increase CD4^+^ T cell transformation into T_regs_ and negatively regulates T-cell-mediated immune responses (Fig. 1), although the exact mechanisms for this are still unclear [74, 75]. Interestingly, examples of synergy between B7-H4 and PD-L1 ICB have been reported [76, 77]. Notably, *EGFR*-mutant NSCLCs express elevated levels of B7-H4 in a MEK/ERK dependent manner [78]. Since ~ 15% of NSCLC patients treated with the KRAS^G12C^ inhibitor sotorasib develop resistance by enhancing EGFR signalling [79] and with clinical benefit already seen in KRAS^G12C^ CRCs [73], these findings suggest that B7-H4 could be a promising ICM target for KRAS^G12C^ inhibitor-resistant NSCLC and CRC, or in combination with KRAS^G12C^ inhibitor, anti-EGFR therapy and PD-L1 ICB. With the first phase I clinical trial of the B7-H4 ICB monoclonal antibody FPA150 (NCT03514121) recently completed [80], there is growing potential for new combination therapies (Table 1).

#### Consequences of aberrant KRAS and PD-L1 signalling on TILs

Oncogenic KRAS modulates both the expression of ICMs and the infiltration of TILs, which are important factors of the anti-tumour immune response. Until recently, there has been less emphasis on investigating the regulation of ICM expression on TILs and how this is influenced by tumour signalling [81]. This issue is complicated as ICM expression on TILs is highly varied between cancers and lymphocyte populations; for example, NSCLC and head and neck squamous cell carcinoma display an increased population of CD45^+^/CD3^+^ TILs while other cancer types have a reduction in this population [81]. However, there are some common themes; a higher proportion of CD3^+^/CD4^+^/CD25^+^/FoxP3^+^ T_regs_ and reduced NK populations are consistently associated with late-stage malignancies, and T cell expression of Tim3 is highly correlated with LAG3 expression, though negatively correlated with CD28 (Fig. 2) [81]. T cells in advanced vs early-stage tumours displayed increased CD244, PD-1, CTLA4, CD39, PD-L2, LAG3, TIGIT, Tim3 and decreased expression of CD73 (Fig. 2) [81].

As with adaptive immunity, KRAS-linked cytokine expression can induce mechanistic alterations in the innate immune system, thus impeding adaptive immune activation and treatment response [82]. For example, *KRAS*-mutation status has been linked to PPARδ-CCL2 expression (involved in M2 macrophage transformation and recruitment) [83, 84], reduced NK cell cytotoxicity by increased expression of HLA-E [85] and PD-L1 [86], and epithelial-mesenchymal transition (EMT) [52], a sensitiser for tumours to NK cytotoxicity (Fig. 2). Indeed, *KRAS*/*TP53* co-mutated tumours promote TNF-driven pro-tumour inflammation [87], and increases in NK-specific MHC complexes (MICA/B) [87, 88], while *KRAS*/*MYC* co-mutation in lung cancer drives the recruitment of anti-inflammatory macrophages and the blocking of NK infiltration by CCL9 and IL-23 (Fig. 1, Fig. 2) [34]. Collectively, these data support the potential for leveraging the innate immune system, particularly NK cells, in the treatment of *KRAS-*mutant tumours. Specific pro-immune mutations have been discovered in patients where NK cells harbour FcγR with the rs396991 genotype (FcγR3a), causing heightened affinity to the IgG1 Fc domain by NK cells and a subsequent increase in IgG1-driven antibody-dependent cell cytotoxicity (ADCC) by NK cells when using IgG1 based mAb therapies such as avelumab [38]. Treatments such as adoptive cell transfer [89] are being explored to fill this niche, although no clinical studies have commenced so far.

#### Metabolic and structural remodelling of the TME by oncogenic KRAS

KRAS drives TME remodelling via several pathways. Enhanced IL-1 and IL-6 expression has been observed in *KRAS*-mutant tumours [34], which leads to upregulation of IDO-1 in TILs via NF-kB and STAT3 signalling (Fig. 2) [90]. This results in a change in metabolism with a subsequent increase in kynurenine production and removal of tryptophan [35], simultaneously causing exhaustion and enhancing PD-1 expression in CD8 + T cells [8], increasing PD-L1 expression in myeloid cells, and stimulating the transformation of CD4^+^ T cells to FoxP3^+^ T_regs_ (Fig. 2) [91]. This is particularly relevant, as the proportion of CD8^+^/PD-1^+^ T cells relative to PD-1^+^ T_regs_ is a predictive biomarker for response to PD-1 blockade [92]. Furthermore, PD-1 expression is negatively correlated with the maturation of macrophages and DCs, and is associated with MDSC and T_H_17 differentiation within the TME (Fig. 2) [93]. T_H_17 are characterised by their production of IL-17, a proinflammatory cytokine that promotes pro-tumour inflammation and inhibits antitumour immunity through the recruitment of both MDSCs and neutrophils into the TME (Fig. 2) [94].

Oncogenic KRAS signalling can lead to increased expression of IL-8, IL-10, GM-CSF, CXCL1, CXCL12, CCL2 and MIF [7, 83], and decreased IL-18 [34], with the subsequent remodelling of the TME to an “immune-cold” contexture, which is more refractory to immunotherapy (Fig. 2). Moreover, secretion of these immune factors influences the recruitment of neutrophils, reduced inflammation via T_H_1 and macrophage inhibition, transformation of macrophages to M2-like TAMs [49, 95], triggering of chemotactic factor expression and MDSC infiltration (Fig. 2) [7, 96]. Such factors have already been targeted therapeutically, with adoptive CAR-T cell transfer in combination with IL-10 blockade being explored to combat resistance to anti-PD-L1 therapy by KRAS-driven cytokine expression [97]. Aberrant cytokine/chemokine signalling in *KRAS*-mutant tumours, such as IL-1β, IL-8, TGF-β and CXCL1 [34, 98], can also result in structural remodelling of the TME extracellular matrix (ECM) by neutrophils, CAFs [99] and M1 macrophages [34] resulting in degradation of ECM by MMP-9 and collagenase IV and the release of bound angiogenic factors such as VEGF [99], as well as cross-linking of ECM collagen fibres by lysyl oxidases (LOX) to enhance matrix stiffness and provide a physical barrier for immune cell infiltration (Fig. 2) [99, 100]. Interestingly, collagen-based discoidin domain receptor 1 (DDR1), CD44 and integrin signalling have been shown to stimulate both the MEK/ERK and PI3K/AKT axis independently of RAS signalling, and cause expression of PD-L1 in response to ECM stiffness (Fig. 2) [98]. Notably, DDR1 inhibition can attenuate *KRAS*-mutant tumour progression [101]. Taken together, the prospect of targeting TME remodelling is evolving to be a potential therapeutic avenue for reversing oncogene-driven immune evasion and the induced “immune-cold” state.

#### Preclinical testing of cancer immunotherapies

Historically, preclinical models for testing ICB have been restricted to highly mutated syngeneic mouse cancer cell lines. Although these have proved incredibly useful, their limited genetic diversity has restricted the number of targeted combinations that can be meaningfully tested in mice. In addition, autochthonous models of RAS-driven tumours have fewer mutations and are not responsive to immunotherapies [39]. Boumelha et al. recently developed an immunogenic *KRAS*-mutant lung cancer model (KPAR) [39] and compared it to a non-immunogenic (KPB6) mouse cancer cell line. Interestingly, expression of human APOBEC3B in *Kras*^*LSL−G12D/*+^*/Trp53*^*fl/fl*^*/Rosa*^*A3Bi*^ mice with *Rag2*^*−/−*^ (KPAR) background failed to produce a TMB/neoantigen-high tumour, but a clonal line from the resulting tumour gave rise to an immunogenic model with MHC-I presentation of the retroviral antigen Emv2; a common tumour antigen in other immunogenic lines (CT26, B16, MC38) [102], as well as human breast cancer and melanoma [103]. When grown in immunocompetent *Rag2*^±^ mice, the KPAR line displayed markers of increased immunogenicity, such as increased infiltration of TILs, PD-L1^+^ myeloid cells, and susceptibility to anti-CTLA-4/anti-PD-L1 combination treatment [39]. This model has been used to demonstrate the importance of IFN-γ in the immune response to *KRAS*-mutant tumours [7]. Of note, orthotopic tumours seeded using the KPAR line displayed differential TIL populations, ICB response and survival rates when compared to tumours initiated by subcutaneous injection, further indicating the similarity between this model and in situ tumours. The importance of orthotopic models in lung [104, 105] and colorectal cancer for the study of immune mechanisms have already been noted [106]. The KPAR model will be a useful addition to LL/2, an alternative mouse tumour model used for lung orthotopic tumour generation, which is extremely immune evasive [107, 108].

### Future prospects for immune evasive KRAS tumours

As new instances of KRAS inhibitor/ICB-induced resistance are uncovered, new treatment opportunities are identified. For example, VISTA is an ICM receptor binding VSIG3 with high sequence and functional homology to PD-1. Elevated expression of VISTA on T cells, MDSCs and TAMs are associated with oncogenic KRAS-driven tumours [109] and resistance to anti-PD-1 by ICB [110, 111], identifying VISTA as a promising immunotherapy target. This was supported by the observation that in an acidic TME, as is common in *KRAS*-mutant tumours due to the enhancement of the Warburg effect [112], VISTA can also bind PSGL-1, another potent inhibitor of T cell activity promoting an exhausted phenotype (Fig. 1) [113]. Recently, the effects of a VISTA mAb (HMBD-002) inhibiting VSIG3 suppression of T cell activity, have been shown to be potentiated by a distinct shift in the TME to a pro-inflammatory phenotype, thus inhibiting tumour growth in vivo in preclinical syngeneic and humanised murine models of colorectal, lung, and breast cancer [114]. Indeed, a clinical trial testing HMBD-002 and pembrolizumab is currently recruiting (NCT05082610), with eagerly awaited results (Table 1). Additionally, preclinical testing of the VISTA-VSIG3/PSGL-1 blocking mAbs BMS-767, and SG7 have demonstrated enhanced T cell activity, though these have not yet progressed to clinical trials [110]. Future prospects for these mAbs potentially include co-treatment with anti-PD-1 ICB and KRAS^G12C^ inhibitors in immunotherapy-resistant NSCLC.

The first KRAS^G12C^ mutant-specific inhibitors provided significant benefit to patients harbouring KRAS^G12C^-driven malignancies such as NSCLC [2, 7]. The most common *KRAS* mutation, G12D, is found in 29.5% of all cancers [15], and 40–50% of PDAC tumours [18, 115]. Recently, potent KRAS^G12D^ -specific inhibitors have been described (TH-Z816 [115] and MRTX1133 [116]). These compounds also exploit the same switch-II pocket as sotorasib but target the mutant aspartic acid. Interestingly, both MRTX1133 and TH-Z816 rely on the formation of a salt bridge rather than covalently bonding to the aspartic acid residue, with TH-Z816 able to inhibit both GDP and GTP-bound forms of KRAS, a mechanism which could potentially be exploited for other mutant-specific KRAS inhibitors [115, 116]. Both compounds have been shown to elicit significant tumour regression in mouse models. Notably, the regression in MRTX1133 was conducted in immunodeficient CD-1 mice [116], while a humanised C57BL/6 mouse model was used for TH-Z618 and also showed synergy with an anti-PD-L1 therapy [115].

Immune escape of tumour cells is often associated with an “immune-cold” and anti-inflammatory TME. As a result of these immunosuppressive mechanisms, and the invasive nature of tumours into surrounding tissues, microorganisms can often be found within tumours giving rise to an intratumoral microbiome, further altering the immune state of the tumour [117]. The effect of the microbiome in *KRAS*-mutant cancers has been recently characterised by Alam et al. [118] where they describe how activation of dectin-1 by the fungal microbiome of KRAS^G12D^ PDAC is crucial for enhanced expression of IL-33, the principal cytokine for the recruitment of innate lymphoid cells 2 (ILC2) into the TME (Fig. 2). This caused increased IL-13 and IL-5 signalling in the TME, resulting in increased M2 macrophage recruitment and immunosuppression; a process which was abated by antifungal treatment [118]. These changes to the TME enhanced tumour immunosuppression via inhibition of NK cells and enhancement of T_regs_ and MDSCs in both lung [119] and colorectal malignancies [120] (Fig. 2). Furthermore, infiltration of the gut microbiome is linked to *TP53* conversion from tumour-suppressor function to oncogenic in CRC [121], a common co-mutation in *KRAS*-mutant tumours. Finally, dysbiosis of the microbiome has been linked with increased ICM expression, such as PD-1/L1 and CTLA-4 in solid tumours [122]. The effect of tumour microbiota on immune evasion in *KRAS*-mutant tumours, and how this is linked to tumour-intrinsic signalling, is an exciting area for future investigation.

### Potential toxicities involved in novel combination therapies

Although combinations of ICB with MAPK pathway inhibitors have the potential to be highly effective, they could also have more serious toxicity profiles. MAPK pathway inhibition is generally associated with gastrointestinal (diarrhoea, colitis) and dermal toxicities (rash, paronychia), with rarer cases of cardio-pulmonary (dyspnea, hypoxia, hypotension) and other toxicities (pyrexia, acute inflammation, neurotoxicity) [123–126]. Toxicities for pan-KRAS inhibitors are unknown, but will likely have widespread activity in normal tissues, similar to MEK and ERK inhibitors [127–129]. Likewise, mTOR inhibitors have gastrointestinal and dermal effects, which could become higher grade in combination [130, 131]. Immunotherapies such as ICB, CAR-T cell, CCR2i and IL-10 blockade primarily present with autoimmune-linked toxicities, due to their effect on self-tolerance. Regular toxicities include acute inflammation (colitis, bowel abscesses, rash), cytokine storm, pyrexia, endocrinopathy, hypotension, and neuropathies [132–137]. MAPK modulators in combination with immunotherapies could worsen gastrointestinal and dermal toxicities specifically, or increase the frequency of rarer effects such as neuropathies. A phase 3 trial (NCT02908672) combining atezolizumab (ant-PD-L1), cobimetinib (MEKi) and vemurafenib (BRAFi), observed toxicities of diarrhoea, nausea, rash and pyrexia, with some participants experiencing acute inflammation, and peripheral neuropathy (Table 1) [138]. Combinations with DDR1i and AXLi, are predicted to have a similar toxicity profile, with diarrhoea, nausea, rash and inflammation common [139, 140]. DDR1 receptors are confined to the epithelial compartment and DDR1 inhibitors also cause inhibition of the MAPK pathway [139], therefore combining DDR1 inhibitors with MAPK inhibitors could lead to more severe toxicity. In summary, the toxicity profiles of monotherapies can inform the probable toxicities of drug combinations, but careful investigation of how these drugs may interact in patients is warranted in order to increase the success rate of these clinical trials.

## Concluding remarks

Our growing understanding of how oncogenic signalling can affect immune evasion strategies has important therapeutic implications in oncology [141]. Targeted therapies can reverse immune evasion programs in *KRAS*-mutant tumours, clearly operating through cell-autonomous and non-cell-autonomous mechanisms [2, 7], which can be enhanced by combination with ICB. If performed correctly, this strategy could partly ameliorate key limitations of either treatment strategy alone—that is, a limited subset of responders in ICB, and the rapid onset of acquired resistance to targeted agents. The research community is developing more therapeutically relevant preclinical models to test these approaches, and we have highlighted a number of exciting ongoing trials to test these strategies in humans (Table 1). More research will be required to investigate how chemotherapies and targeted therapies influence other important factors, such as TMB [30], intratumoural heterogeneity [142], and the host/tumour microbiome. In the future, a deeper understanding of how the specific constellation of genetic drivers in a patient’s tumour influences the TME will ultimately improve precision medicine approaches and responses to immunotherapies.

## Data Availability

All data within this review is fully referenced and publicly available through the referenced unique DOI.
